# The effect of socioeconomic status, depression, and diabetes symptoms severity on diabetes patient’s life satisfaction in India

**DOI:** 10.1038/s41598-024-62814-5

**Published:** 2024-05-28

**Authors:** Shubham Ranjan, Ramna Thakur

**Affiliations:** School of Humanities and Social Sciences, Indian Institute of Technology Mandi, Mandi, India

**Keywords:** Diabetes, Patients, Socioeconomic status, Life satisfaction, Depression, Structural equation modelling, Diseases, Endocrinology, Signs and symptoms

## Abstract

Evidence suggests that diabetes is on the rise in India, affecting many people’s life satisfaction. Comprehensive estimation of life satisfaction among diabetes patients does not exist in the country. This study examined the effects of socioeconomic status, depression, and diabetes symptoms severity on the life satisfaction of diabetes patients by controlling various demographic variables. It was a cross-sectional study comprising 583 diabetes patients from Punjab, India. Patients were interviewed using a multi-stage purposeful random sampling method. Descriptive analysis and partial least squares structural equation modelling were used in the study to test the hypotheses. Results revealed that socioeconomic status, depression and diabetes symptoms severity significantly influence the life satisfaction of diabetes patients. A 1% drop in diabetes symptoms severity corresponds to a 0.849% increase in life satisfaction, whereas a 1% decrease in depression results in a 0.898% increase in life satisfaction. Patients with higher diabetes symptoms severity were coping with common mental disorders. Women reported higher diabetes symptoms severity and depression than men, resulting in lower life satisfaction. An experimental evaluation of the effects of socioeconomic status, depression and diabetes symptoms severity, and numerous demographic factors on life satisfaction was reported. The findings will help policymakers understand the problem associated with life satisfaction among diabetes patients in the country.

## Introduction

Diabetes, being a chronic disease, significantly impacts health and is a major global public health concern^1,2^. It is one of the main contributors to mortality and morbidity worldwide, especially in India^3^. India is known to be the “Diabetes Capital of the World”, with around 19.4 million people living with diabetes, and by 2025, that figure is anticipated to ascend to 57.2 million^4,5^. Such an increasing burden and the chronic nature of this disease has a multidimensional impact on the individual, family, and society^6^. Its multifaceted impact has been linked to several physical, psychological, and social issues that require substantial and ongoing assistance from various societal stakeholders^7,8^. Several studies have found that these issues are significantly associated with life satisfaction (LISAT) in diabetes patients^9–13^.

In this direction, Tuncay and Avcı^14^ in their study found that the level of LISAT varies across diabetes patients. Evidence suggests that LISAT and diabetes management are interconnected^15^. As a result, in the context of diabetes, LISAT, one of the oldest multidimensional concepts, appears to be more complicated than it seems^16^. Generally, its measures are subjective or based on the variables an individual finds personally crucial^17^. For instance, many studies have found that diabetes patients with a high LISAT have more fulfilling social relationships, receive more social support, and feel more satisfied than those with a low LISAT^18–20^.

Additional studies conducted in this area have shown that a higher LISAT is positively related to better diabetes management and helps reduce its risk factors among patients^15,21–23^. Patients with a high LISAT have a significantly lower risk of mortality than their low LISAT counterparts^24–28^. Furthermore, the low LISAT levels among patients with diabetes compared to the general population may reflect an additional burden on them^19,20^. With the increasing burden of diabetes, it is crucial to understand how patients’ lives are affected and the factors influencing their LISAT.

Studies conducted by various researchers, such as Baumann et al.^14^ in Luxembourg, Mauricio^18^ in Spain, and Tuncay and Avcı^21^ in Turkey, have shown positive results in diabetes patients with decent LISAT. A study conducted by Lee et al.^19^ in Taiwan suggests that improvements in LISAT positively impact diabetes management and overall mental well-being. Thus, a comprehensive study that examines the relationship between LISAT, diabetes and its related factors is important for developing countries like India. In a similar direction, this study included diabetes symptom severity (DSS) and its relationship with depression in the case of diabetes patients, and, ultimately, their LISAT. Furthermore, we have examined the effects of diabetes patients’ socioeconomic status (SES) on DSS, depression, and LISAT while controlling for gender, age, resident type, religion, social category, and household size. By gaining a deeper understanding of these factors, healthcare professionals and policymakers can develop targeted interventions and support systems to improve the LISAT of patients with diabetes. Such understanding can help them in developing effective strategies to support patients with diabetes and improve their overall well-being in the country.

### Aims and objectives of the study

The LISAT measurement scale includes many essential domains such as vocational, financial and leisure situations, contacts with friends, sexual life, self-care management, family life, partner relationships, and physical and psychological health^29^. Evidence shows that the effects of each LISAT domain are unequal and vary between individuals^30^. Thus, there is a need to investigate the effect of potential factors that may significantly influence LISAT in diabetes patients^31^. As a result, our study aimed to assess the effect of SES, depression, and DSS on the LISAT of diabetes patients. An additional aim was to identify the potential mediating role of depression and DSS in the relationship between SES and LISAT. Both analyses were conducted while controlling for numerous demographic variables.

## Methodology

### Measures

This section briefly outlines the constructs and associated indicators used in our study. We have also discussed the development of measures used in survey instruments. The measures adopted in the present study are well-established and widely used scales in the literature. However, the indicators were modified as per the objectives of the study.

#### Socioeconomic status (SES)

SES is a complex concept that includes the living conditions, resources, and opportunities available to people within a particular society. It measures one’s combined economic and social status, often associated with health^32^. Therefore, from a measurement perspective, it is conventionally conceptualised as a formative latent variable rather than a reflective latent variable^33^. Thus, we employed several observable factors (Supplementary File 1) to establish SES as a formative latent construct while considering India’s demography^34,35^.

#### Life satisfaction (LISAT)

We used the LISAT-11 questionnaire to measure the overall LISAT level of diabetes patients. It is an extension of LISAT-9, generally treated as a reflective latent construct where several studies have employed it to assess the LISAT of different patients^36–38^.

#### Patient health questionnaire (PHQ)

The PHQ is a reflective latent construct used to assess common mental disorders in various pieces of research^39^. In the case of diabetes, this simple self-administered diagnostic instrument has been used extensively to detect the early stages of depression^40–45^.

#### Diabetes symptoms severity (DSS)

The DSS is constructed as a reflective latent construct based on a self-administered questionnaire designed to gauge how severe diabetes patients perceive their symptoms. The questionnaire included a list of symptoms of diabetes which have been taken from the medical literature, and the severity of each symptom was determined using a five-point Likert scale, with scores ranging from 0 (not present) to 4 (most severe)^46^. This questionnaire not only assesses perceived DSS but also examines the chronic progression of the disease. Since many people usually ignore the symptoms of diabetes for a substantial period and sometimes do not consider them serious because, unlike many other health conditions, the effects of diabetes does not appear immediately^47^. People are generally unaware that damage can begin many years before symptoms become noticeable^48^. Hence, by considering the asymptomatic nature of diabetes in the early stages, it is crucial to measure the chronic progression of diabetes because early recognition of symptoms can help patients get the disease under control sooner and provide protection against vascular complications^49^.

#### Control variable

In this study, Table 1 shows the demographic profiles (DP) such as resident type (DP1) (rural and urban), age group (DP2) (19–50 years, 51–60 years and 61–above years), religion (DP3) (Sikh and others including Hindu and Muslim), social category (DP4) (others and SC & OBC), gender (DP5) (women and men), and household size (DP6) (Below average and Above average) with average of 4.83 (≈ 5) persons. Previous studies have shown the importance of these variables that may affect model outcomes^16,23,50,51^.

**Table 1:** Demographic summary.

Characteristics	Types	Total cases (Percentage)
Demographic profile (DP)		
Resident type (DP1)	Rural	324 (55.6)
	Urban	259 (44.5)
Age group (µ = 58.5 years) (DP2)	19–50	169 (29.0)
	51–60	172 (29.6)
	61-above	242 (41.6)
Religion (DP3)	Others (Hindu, Muslim)	210 (36.1)
	Sikh	373 (64.0)
Social category (DP4)	Others	368 (63.2)
	SC and OBC	215 (36.9)
Gender (DP5)	Women	299 (51.3)
	Men	284 (48.8)
Household size (µ = 4.83 person) (DP6)	Below µ	415 (71.2)
	Above µ	168 (28.9)

µ = Mean.

### Model building and hypothesis development

#### Diabetes and SES

Years of research have shown that SES affects diabetes disproportionately, where relatively consistent patterns are observed in the risk and prevalence of diabetes and its complications among different levels of SES^52–54^. Despite the interdependence of the components of SES, each has a distinct effect on diabetes. For instance, the complications and prevalence of diabetes vary significantly by the level of education and wealth^55,56^. However, on a broad scale, people with lower SES levels are more likely to develop diabetes, have more complications, and die earlier than those with higher SES levels^57–59^. Variations in SES pose unique challenges for diabetes patients in lower-middle-income nations like India. Such anomalous variation in SES across countries differentially affects the risk factors, prevalence, and burden of diabetes^60^.

#### SES and LISAT

Literature shows that people with high SES are better at controlling their diabetes than those with low SES^61^. Many qualitative and quantitative research studies has also demonstrated a positive association between SES and LISAT levels^62–64^. Accordingly, increasing LISAT is recommended not only for people with diabetes but also for the general population. Several studies have proved that a higher LISAT slows down the disease progression rate^65,66^. The study of Carniglia et al.^67^ suggests that strengthening SES can improve LISAT, which is critical in the case of diabetes as well.

#### SES and DSS

The major problem for any diabetes patient is managing the symptoms and the severity associated with the same^68^. As a result, proper diabetes management necessitates balancing the SES level of the patient^69^. In this direction, several studies have found SES to be a central factor in overall diabetes management, including its symptom management and associated complications^57,70,71^.

#### SES and PHQ

SES is not only linked with diabetes management but is also significantly associated with other complications of diabetes, such as depression, obesity, hypertension, etc.^60,72^. Furthermore, Everson et al.^73^ found that the least affluent bear a disproportionate amount of the disease burden, including depression, obesity and diabetes. Likewise, Leone et al.^72^ corroborated that a low SES is associated with a higher prevalence of depression among diabetes patients.

#### PHQ and LISAT

In a similar line, the level of depression among diabetes patients also has an impact on their LISAT levels^19^. A study by Nigeria et al.^23^ found that more depressed patients reported lower LISAT and vice versa. LISAT is not only associated with depression among patients with diabetes but also with complications perceived by patients due to diabetes symptoms^47^.

#### DSS and LISAT

Tekir et al.^74^ conducted a study on the effect of diabetes symptoms on LISAT and found that diabetes symptoms affect LISAT. The findings of Kim and Lee showed that diabetes patients with andropause symptoms experienced a low level of LISAT^75^. Further, Gulliford and Mahabir^71^ found that DSS has a negative impact on health-related quality of life. Hence, raising LISAT among diabetes patients is recommended by several studies^14–16^. A high level of LISAT is vital in keeping diabetes and its complications under control^14^.

#### DSS and PHQ

Further, earlier research has found that diabetes patients who have their diabetes-related problems under control are less likely to suffer from depression^9^. However, because most patients are unable to control their diabetes, thus, diabetes and depression occur together almost twice as often as would be predicted by chance alone^76^. Grootenhuis et al.^77^ found substantial differences in symptom severity scores among patients with diabetes to be the leading cause of depression among patients. In a similar direction, Katon^78^ showed an overlap between diabetes symptoms and depression. Hence, many studies have proven that the aforementioned highlighted components are essential for further analysis in the case of diabetes patients^69,72,74^.

**Figure 1:**
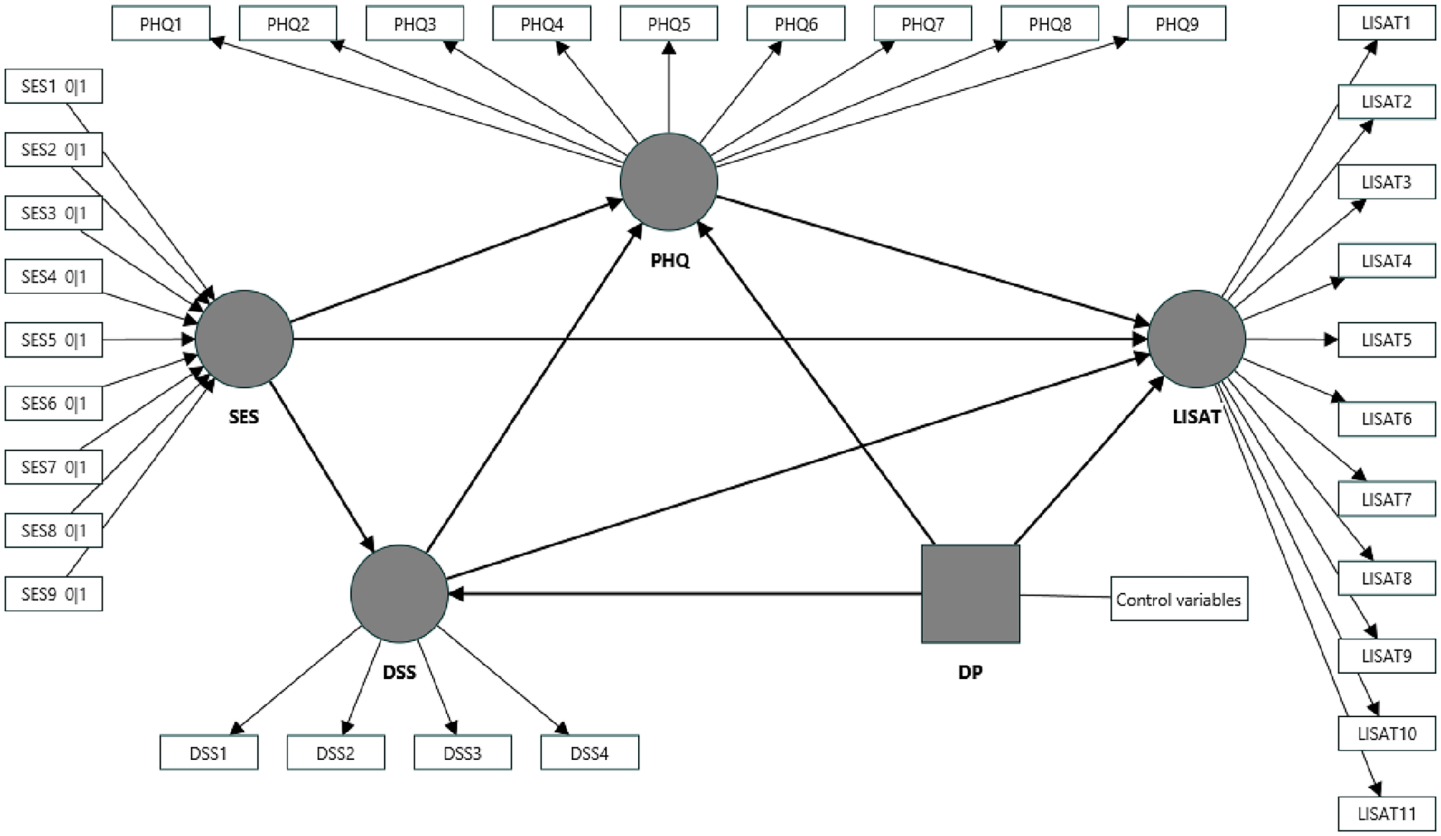
SEM model specification. Control variables include gender, age, resident type, religion, social category, and household size.

All these studies together helped in developing the following hypothesis in the present study (see Fig. 1):

##### H_1_

SES has a positive relationship with LISAT among diabetes patients.

##### H_1a_

DSS mediates the relationship between SES and LISAT.

##### H_1b_

PHQ mediates the relationship between SES and LISAT.

##### H_1c_

DSS and PHQ mediate the relationship between SES and LISAT.

##### H_2_

SES has a negative relationship with DSS among diabetes patients.

##### H_3_

SES has a negative relationship with PHQ among diabetes patients.

##### H_3a_

DSS mediates the relationship between SES and PHQ.

##### H_4_

DSS has a negative relationship with LISAT among diabetes patients.

##### H_4a_

PHQ mediates the relationship between DSS and LISAT.

##### H_5_

PHQ has a negative relationship with LISAT among diabetes patients.

##### H_6_

DSS has a positive relationship with PHQ among diabetes patients.

#### Data and sampling methods

The present paper was based on primary cross-sectional data of diabetic patients collected through well-structured questionnaires from different districts of Punjab, India. All the data on diabetes-related symptoms were collected during diabetes, where the average duration of diagnosis was 4.75 years. A sample of households for collecting the required information was selected with the help of a multi-stage purposeful random sampling method^79^. We followed the WHO-STEPS methodology to prepare, design, and select the samples in the study area and used the census of 2011 as the sampling frame^80,81^. The target population in the study area was patients who had diabetes. The survey period was from November 2020 to February 2021 and from March 2021 to June 2021. This study was performed in line with the principles of the Declaration of Helsinki. The ethical clearance approval was obtained from the INSTITUTE ETHICAL COMMITTEE (HUMAN) of the Indian Institute of Technology Mandi, India (REF: IITM/IEC(H)/2022/RT/P1).

### Statistical analysis

We have performed descriptive statistics such as frequency and percentage distribution and partial least squares structural equation modelling (PLS-SEM) using the SmartPLS4 version to calculate the results^82^. PLS-SEM allows the estimation of complex cause-effect relationships in path models with latent variables^83^.

### Ethics approval

Approval was granted by the Institutional Ethics Committee of the Indian Institute of Technology Mandi on 12/01/2022 (REF: IITM/IEC(H)/2022/RT/P1).

### Consent to participate

Data was collected after obtaining the informed consent from the patients. Field enumerators explained the purpose of the data collection to the subjects and obtained their approval before proceeding with the data collection.

### Human and animal rights

This study was conducted in accordance with the principles of the Declaration of Helsinki. The authors did not conduct any biological experiments on human or animal subjects in this study.

## Results

Table 1 shows the sample distribution of 583 diabetes patients across various demographic factors, consisting of 299 (51.3%) women and 284 (48.7%) men, categorised into three age groups: 19–50 years, 51–60 years and 61–above years, where the mean age was 58.5 years. Our results showed that the proportion of diabetes patients increased as we moved from lower to higher age groups. In our study, 55.6% of patients were from rural areas, and 44.4% were from urban areas. Also, the majority of the patients (64.0%) belonged to the Sikh community, as the state has a substantial Sikh population, accounting for 57.7% of the total population of Punjab. Furthermore, the average household size in our study was 4.83 (≈ 5) persons, which was higher than India’s average of 4.44 (≈ 4) persons.

### Evaluation of reflective measurement model

Many studies highlighted the importance of reflective measurement modelling in SEM^84–87^. Therefore, it is crucial to perform its assessment before inclusion in the final model^88^. The primary goal of such an evaluation is to ensure the reliability and validity of the reflective construct measures and consequently provide support for the suitability of their inclusion in the path model. As per the criteria stated by Hair et al.^89^, Cronbach’s alpha and composite reliability values should lie between 0.700 and 0.950. Whereas the average variance extracted (AVE) value should be at least 0.50, indicating that the construct explains 50% or more of the variance of the indicators that make up that particular construct. Similarly, the results presented in Table 2 passed the threshold, confirming that reliability and convergent validity were achieved for the LISAT, PHQ, and DSS.

**Table 2:** Reflective construct reliability and validity analysis.

Reflective Constructs	Items	Outer loadings	Variance inflation factor (VIF)	Cronbach’s alpha	Composite reliability (rho_c)	Average variance extracted (AVE)
LISAT	LISAT1	0.935	1.873	0.921	0.930	0.563
	LISAT2	0.443	2.308			
	LISAT3	1.039	3.342			
	LISAT4	1.053	4.051			
	LISAT5	1.084	2.821			
	LISAT6	0.954	1.950			
	LISAT7	0.817	2.487			
	LISAT8	1.041	3.551			
	LISAT9	0.373	2.764			
	LISAT10	0.879	3.493			
	LISAT11	1.152	4.007			
PHQ	PHQ1	1.043	1.961	0.926	0.939	0.631
	PHQ2	1.417	2.727			
	PHQ3	1.289	3.497			
	PHQ4	1.153	3.543			
	PHQ5	1.020	2.943			
	PHQ6	0.847	2.321			
	PHQ7	1.026	2.954			
	PHQ8	0.958	2.231			
	PHQ9	0.515	1.651			
DSS	DSS1	0.568	1.180	0.708	0.817	0.535
	DSS2	0.893	1.477			
	DSS3	1.180	1.729			
	DSS4	1.223	1.585			

After confirming the reliability and convergent validity of the LISAT, PHQ and DSS, it was necessary to establish their discriminant validity (DV) for further evaluation. Evaluating DV evaluates construct specificity, which ensures the discrimination between the measures of dissimilar constructs. Hence, to ensure that the construct is unique from the other constructs in terms of empirical criteria, Hair et al.^89^ presented two measures of DV, i.e., through analysing the cross-loadings of the indicators and confirming the Fornell–Larcker criterion (FLC). The cross-loading results in Table 3 show higher values in the diagonal line, confirming the DV where outer loadings of LISAT indicators were higher on the LISAT only. Similarly, PHQ indicators were also higher only on the PHQ. In addition, DSS indicators were high only for the DSS and not for the LISAT and PHQ, respectively. Concurrently, in Table 3, the FLC results show that the shared variance among LISAT, PHQ and DSS in the model did not exceed their AVE. In empirical applications, however, FLC consistently fails to reliably identify DV issues because it performs insufficiently, mainly when indicator loadings vary slightly over a construct. Thus, Henseler et al.^90^ stated that FLC and cross-loading criteria are inadequate methods to ensure DV, and recommended the heterotrait-monotrait (HTMT) ratio for further validation. Accordingly, in our case, the value of the HTMT ratio is below the threshold level of 0.85, indicating that the DV has been established and the model is reliable for further processing.

**Table 3:** Cross loadings, Fornell–Larcker criterion and Heterotrait-monotrait ratio (HTMT).

	LISAT	PHQ	DSS
Cross loadings			
LISAT1	**0.698**	− 0.278	− 0.611
LISAT2	**0.340**	− 0.035	− 0.018
LISAT3	**0.826**	− 0.368	− 0.425
LISAT4	**0.878**	− 0.419	− 0.600
LISAT5	**0.834**	− 0.420	− 0.561
LISAT6	**0.684**	− 0.215	− 0.356
LISAT7	**0.738**	− 0.353	− 0.393
LISAT8	**0.881**	− 0.437	− 0.485
LISAT9	**0.408**	− 0.003	0.035
LISAT10	**0.832**	− 0.396	− 0.426
LISAT11	**0.890**	− 0.418	− 0.555
PHQ1	− 0.358	**0.748**	0.165
PHQ2	− 0.329	**0.772**	0.080
PHQ3	− 0.349	**0.821**	0.096
PHQ4	− 0.419	**0.875**	0.136
PHQ5	− 0.392	**0.838**	0.175
PHQ6	− 0.360	**0.792**	0.083
PHQ7	− 0.401	**0.846**	0.162
PHQ8	− 0.371	**0.768**	0.075
PHQ9	− 0.288	**0.667**	0.002
DSS1	− 0.327	− 0.033	**0.516**
DSS2	− 0.364	0.052	**0.719**
DSS3	− 0.434	0.091	**0.803**
DSS4	− 0.607	0.219	**0.843**
Fornell–Larcker criterion			
LISAT	0.750		
PHQ	− 0.460	0.794	
DSS	− 0.617	0.143	0.731
Heterotrait-monotrait ratio			
PHQ	0.443		
DSS	0.653	0.165	

Factor loadings for each observed variable are in bold.

### Evaluation of formative measurement model

After evaluating LISAT, PHQ and DSS, the results in Table 4 show the evaluation of SES. As previously stated, SES is a formative measurement model; hence, the internal consistency perspective that supports LISAT, PHQ, and DSS assessment cannot be used in the case of SES. Since its measures do not always covary, thus its evaluation starts with convergent validity (CV). CV ensures that the entire domain of the SES and its significant attributes are covered by its indicators. As a result, we performed a redundancy analysis to ensure the CV of SES. Next, we examined the collinearity issue between indicators of SES using the Variance Inflation Factor (VIF), and the results for all indicators were below a threshold of five.

**Table 4:** Formative construct assessment.

Formative Constructs	Indicators	Convergent validity	Variance inflation factor (VIF)	Outer weights (*p*-values)
SES	SES1	Achieved using redundancy analysis	1.230	0.295 (0.000)*
	SES2		1.030	0.361 (0.000)*
	SES3		1.155	0.414 (0.000)*
	SES4		1.054	0.384 (0.000)*
	SES5		1.234	0.404 (0.000)*
	SES6		1.275	0.277 (0.000)*
	SES7		1.292	0.219 (0.000)*
	SES8		1.179	0.198 (0.000)*

*Significant at 5%.

Additionally, considering significant values of outer indicator weights ensures the validation and inclusion of SES in the final model^91^. We may conclude from the results of Tables 2, 3, and 4 that LISAT, PHQ, DSS and SES have a sufficient degree of measurement quality, indicating that all constructs passed the evaluation criteria presented by Hair et al.^89^. Thus, it is recommended to proceed with the evaluation of the structural model.

### Evaluation of structural model

After establishing the outer model, Table 5 shows the results of the proposed hypotheses testing, where we found that SES plays a vital role in coping with DSS and PHQ. Further, the outcomes revealed that DSS and PHQ were negatively associated with LISAT. It implies that a 1% decrease in the DSS of diabetes patients brings a 0.849% positive change in their LISAT, while a 1% decrease in the PHQ improves the LISAT of diabetes patients by 0.898%. Additionally, diabetes patients who perceived higher symptom severity were more depressed. Similarly, our indirect path analysis demonstrated an instance of serial mediation, with DSS as a mediator variable impacting the path from SES to LISAT more than PHQ. Because the variance accounted for by DSS and PHQ were 50.73% and 30.46%, respectively, showing a case of partial mediation in our model.

**Table 5:** Hypotheses testing results.

Effect	Original sample	Sample mean	STDEV	T statistics	*p*-values	Decision
Direct effect						
SES—> LISAT	0.135	0.149	0.111	1.215	0.224	Not supported
SES—> PHQ	− 0.280	− 0.272	0.095	2.936	0.003*	Supported
SES—> DSS	− 0.492	− 0.489	0.186	2.643	0.008*	Supported
DSS—> LISAT	− 0.849	− 0.844	0.054	15.672	0.000*	Supported
PHQ—> LISAT	− 0.898	− 0.895	0.070	12.779	0.000*	Supported
DSS—> PHQ	0.046	0.044	0.029	1.606	0.108**	Supported
Indirect effect						
SES—> DSS—> LISAT	0.418	0.413	0.161	2.592	0.010*	Supported
SES—> PHQ—> LISAT	0.251	0.244	0.088	2.844	0.004*	Supported
SES—> DSS—> PHQ—> LISAT	0.020	0.019	0.014	1.424	0.154	Not supported
SES—> DSS—> PHQ	− 0.023	− 0.021	0.017	1.342	0.180	Not supported
DSS—> PHQ—> LISAT	− 0.042	− 0.038	0.024	1.700	0.089**	Supported

*Significant at 5%, **Significant at 10%.

Results in Table 6 show the significance of control variables in our structural model. We found a significant difference in DSS among resident type and age. Patients in rural areas perceived more DSS than their urban counterparts, whereas, with increased age, patients perceived lower DSS. In the case of religion and social category, Sikh and other category patients reported significantly lower levels of LISAT. Compared to men, women patients had significantly more discomfort from DSS, and their high levels of depression may have contributed to their poorer levels of LISAT.

**Table 6:** Effect of control variables.

Effect	Original sample	Sample mean	STDEV	T statistics	*p*-values
DP1—> LISAT	0.067	0.067	0.073	0.921	0.357
DP1—> PHQ	0.027	0.029	0.035	0.647	0.518
DP1—> DSS	0.183	0.182	0.063	2.920	0.004*
DP2—> LISAT	0.015	0.015	0.041	0.359	0.719
DP2—> PHQ	− 0.005	− 0.006	0.023	0.231	0.817
DP2—> DSS	− 0.089	− 0.089	0.035	2.563	0.010*
DP3—> LISAT	0.128	0.131	0.078	1.645	0.100**
DP3—> PHQ	0.052	0.053	0.043	1.232	0.218
DP3—> DSS	− 0.009	− 0.008	0.066	0.134	0.893
DP4—> LISAT	− 0.111	− 0.112	0.068	1.634	0.102**
DP4—> PHQ	− 0.025	− 0.023	0.043	0.578	0.563
DP4—> DSS	0.103	0.101	0.060	1.723	0.085**
DP5—> LISAT	− 0.085	− 0.086	0.069	1.232	0.218
DP5—> PHQ	0.057	0.055	0.037	1.514	0.130
DP5—> DSS	0.175	0.175	0.054	3.212	0.001*
DP6—> LISAT	0.012	0.008	0.095	0.129	0.898
DP6—> PHQ	− 0.075	− 0.076	0.061	1.239	0.216
DP6—> DSS	0.157	0.157	0.081	1.934	0.053*

*Significant at 5%, **Significant at 10%.

The findings in Table 7 show the structural model’s predictive relevance results. We have used f-square, adjusted R-square, and Q-square values and calculated them for (before and after introducing) the control variables to check the predictive relevance of the structural model. VIF values for all the constructs were less than five, indicating that our model has no collinearity issues. Further, the results of the adjusted R-square (with control variable) for PHQ (0.075) and DSS (0.103) were less than 0.25, implying a weak explanatory power. In contrast, we found a substantial explanatory power in the LISAT (0.518) case because the value was more than 0.50. It means that the model’s independent variables explain 51.8% of the variability observed in LISAT. In addition, we used f-square to measure the effect of any exogenous variable on R-square when an exogenous variable is removed from the model. The analysis of the f-square (with control variable) shows that both PHQ (0.263) and DSS (0.553) have a medium and large effect on LISAT, respectively. The f-square (with control variable) for DSS (0.005 < 0.020) has a small effect on the PHQ, implying that dropping DSS would have little impact on the explanatory power of the PHQ. The results of the Q-square value for all reflective endogenous latent variables were greater than zero, indicating that our values were well reconstructed and that the model had achieved predictive relevance.

**Table 7:** Predictive relevance using f-square, adjusted R-square, Q-square analysis and Cross-validated predictive ability test (CVPAT).

	LISAT f-square without control variable (with control variable) [VIF]	PHQ f-square without control variable (with control variable) [VIF]	DSS f-square without control variable (with control variable) [VIF]	Adjusted R-Square without control variable	Adjusted R-Square with control variable	Q-Square without control variable	Q-Square with control variable
LISAT				0.522	0.518	0.926	0.928
PHQ	0.265 (0.263) [1.096]			0.075	0.075	0.210	0.208
DSS	0.589 (0.553) [1.134]	0.006 (0.005) [1.128]		0.070	0.103	0.741	0.746
SES	0.003 (0.004) [1.189]	0.059 (0.055) [1.127]	0.077 (0.078) [1.046]				

		Average loss difference		t-values		*p*-values	
Cross-validated predictive ability test (CVPAT)							
CVPAT without control variable							
LISAT		− 0.060		2.259		0.024*	
PHQ		− 0.014		2.514		0.012*	
DSS		− 0.028		2.538		0.011*	
Overall		− 0.038		2.624		0.009*	
CVPAT with control variable							
LISAT		− 0.072		2.386		0.017*	
PHQ		− 0.014		2.159		0.031*	
DSS		− 0.044		2.925		0.004*	
Overall		− 0.045		2.809		0.005*	

*Significant at 5%.

In addition to the above analysis, Liengaard et al.^92^ extended the model’s predictive relevance using a cross-validated predictive ability test (CVPAT). CVPAT applies an out-of-sample prediction approach to calculate the structural model’s prediction error. This method also calculates the average loss value of PLS-SEM, compared with the average loss value of the indicator averages and the linear model, which are used as naive and conservative benchmarks, respectively. To substantiate better predictive capabilities of the model compared to the prediction benchmarks, CVPAT tests whether PLS-SEM’s average loss is significantly lower than the average loss of the benchmarks^93^. Therefore, results in Table 5 show that PLS-SEM’s average loss with and without control variables was significantly lower than the average loss of the benchmarks. A negative difference in the average loss values implies that our model has better predictive capabilities.

### Discussion and policy implications

Two main theories about the LISAT exist in the current literature, namely, the bottom-up and top-down theories, first distinguished by Diener^94^. Bottom-up theories consider LISAT to be summary judgments of satisfaction with important life domains, while top-down theories assume that LISAT has a global halo effect on satisfaction with specific life domains^95^. In other words, the bottom-up theories of LISAT are based on the idea that LISAT is the sum of its parts overall. In contrast, top-down theories rest on the premise that satisfaction with domains of life is mainly a consequence of overall LISAT^96^. We have observed that both bottom-up and top-down theories face causality dilemmas: the problems with sorting time sequences. Despite such claims to the contrary, Tuncay and Avcı^14^ found that LISAT is a significant predictor of diabetes. Therefore, given the increasing burden of diabetes, it is recommended to use a mixed approach to study the causes of LISAT, and through causation, linking its multidimensional effects is suggested by several studies^5,18^. As a result, like many studies, we used a bottom-up approach to interpret LISAT and analysed its multivariate effects using a top-down approach for diabetes patients^15,16^.

Our results revealed that SES positively impacts LISAT, PHQ and DSS among diabetes patients in India. In the study area, a 1% rise in SES will improve diabetes patients’ LISAT, PHQ and DSS scores by 0.135%, 0.492%, and 0.280%, respectively. Patients with higher SES receive more diabetes care and can better manage their complications, improving their mental health and LISAT. Strengthening the SES level of the patient through its various domains helps patients cope with diabetes-related issues and reduces the overall burden on society^97^. Consequently, the study by Baumann et al. and Houle et al. recommends that strengthening patients’ SES can improve outcomes in diabetes patients^21,98^.

The outcomes showed that PHQ and DSS were negatively associated with LISAT. This implies that fragmented health policies nationwide and a lack of government support for diabetes patients prevent them from achieving high LISAT^99^. According to Gwozdz and Sousa-Poza’s ^100^ study, a higher LISAT not only enables diabetes patients to recover more quickly, preserve their lifespan and be more productive, but it also reduces the overall burden on the nation. However, India has limited diabetes treatment facilities and effective management programs, which accounts for the significant differences between LISAT, PHQ and DSS among diabetes patients^9,101,102^.

Furthermore, the direct effect results with control variables revealed that patients residing in rural areas perceived higher DSS than their urban counterparts. Deepa et al.^103^ found that knowledge and awareness about diabetes in India, particularly in rural areas, is poor compared to urban areas. At the same time, the availability and utilisation of healthcare services and diabetes-related complications vary between rural and urban areas^104–106^. This means that rural-specific diabetes-care planning needs to be strengthened in the study area. Besides, we found that patients with increased age perceived lower discomfort due to DSS. A study on symptoms by Tibblin et al.^107^ showed that self-reported symptoms generally decreased with age. In addition, biological changes that occur with ageing affect patients’ personal and social responses, which may lead to under-reporting symptoms’ severity^108^.

The importance of social constructs such as gender and their significant association with diabetes, LISAT and PHQ has also been highlighted by several studies^51,109,110^. Similarly, our study revealed a gender difference in all the endogenous variables (LISAT, PHQ and DSS), with women perceiving greater DSS and PHQ than men, which may lower their LISAT. As a result, Kim et al.^50^ suggest that gender disaggregated policy may provide an opportunity to increase LISAT. In line with our results, the importance of studying socioeconomic and demographic aspects in reducing the burden of diabetes has been highlighted by the study of Wang et al.^111^. Our research will thus assist policymakers in concentrating on those patients who actually require improvement. The results of the study may contribute to better healthcare delivery for such a large number of diabetes patients, especially those whose LISAT is affected by SES, DSS and depression.

## Conclusion

This article explores the underlying association between SES and LISAT through a lens of PHQ and DSS, examining their multidimensional impact across various demographic variables. The present study’s data was collected from diabetes patients in Punjab, India, and PLS-SEM was used to test multiple hypotheses. The results showed that SES was the primary factor affecting LISAT, PHQ and DSS in the case of diabetes patients. Therefore, improving the SES of patients can lead to an overall improvement in disease management and help reduce the overall disease burden in the country. This implies that our study has practical implications for policymakers, which may assist them in doing interventions related to SES, LISAT, PHQ and DSS, perhaps resulting in multidimensional benefits to diabetes patients in India.

Additionally, we have identified breathtaking possibilities for future research in this area. Although this paper focused only on Punjab, India, it would be worthwhile to consider LISAT predictions in different states of India and various countries using cross-national data. The availability of panel data would make it possible to control unseen heterogeneity and move towards making predictions about causal relationships between LISAT and SES in the case of diabetes patients. Furthermore, this study's scope could extend to other chronic diseases such as cancer, cardiovascular diseases, etc. Since we found little research on LISAT predictors of such health conditions where the existing studies have been conducted in developed countries. Nevertheless, we found some evidence about the importance of SES in interpreting the LISAT in the literature, but their context was narrow as opposed to broad measures of SES. Despite several limitations, this study provides valuable insights for policymakers to conduct comprehensive studies on such predictors, which will help them control the disease burden and improve the overall LISAT of the patients in the country.

### Supplementary Information

Supplementary Information.

## Data Availability

The datasets generated during and/or analysed during the current study are available from the corresponding author upon reasonable request.
