# Supplementary material for: The effect of socioeconomic status, depression, and diabetes symptoms severity on diabetes patient’s life satisfaction in India
Source: Sci Rep. 2024 May 28;14:12210. doi: 10.1038/s41598-024-62814-5 (PMC11133318; doi:10.1038/s41598-024-62814-5)
Supplement: Supplementary file 1 — Supplementary Information. [file 41598_2024_62814_MOESM1_ESM.docx]

**Supplementary File 1: Construct summary**

| **Characteristics** | **Types** | **Total cases (Percentage)** |
| --- | --- | --- |
| **Socio-economic status (SES)** | | |
| Educational level (SES1) | Illiterate | 182 (31.2) |
|  | Literate | 401 (68.8) |
| Type of work (SES2) | Domestic work | 242 (41.5) |
|  | Income generating | 341 (58.5) |
| Per person monthly expenditure (SES3) (µ = ₹4766.41) | Below µ | 350 (60.0) |
|  | Above µ | 233 (40.0) |
| Size of the land holding (in hectare) (SES4) | Only residence | 403 (69.1) |
|  | Residence with land | 180 (39.9) |
| Water Purifying System (SES5) | Nonelectric | 309 (53.0) |
|  | Electric | 274 (47.0) |
| Primary source of cooking (SES6) | LPG | 517 (88.7) |
|  | No LPG | 66 (11.3) |
| Separate kitchen (SES7) | No | 55 (9.4) |
|  | Yes | 528 (90.6) |
| Household material (SES8) | Concrete | 536 (91.9) |
|  | Mixed | 47 (8.1) |
| Type of latrine (SES9) | Flush system | 272 (46.7) |
|  | No flush system | 311 (53.3) |
| **Life-Satisfaction (LISAT)** | | |
| Ability to manage self-care (dressing, hygiene, transfers, etc.) (LISAT1) | Very dissatisfying | 45 (7.8) |
|  | Dissatisfying | 101 (17.4) |
|  | Rather dissatisfying | 179 (30.7) |
|  | Rather satisfying | 59 (10.2) |
|  | Satisfying | 106 (18.2) |
|  | Very satisfying | 93 (16.0) |
| Financial situation (LISAT2) | Very dissatisfying | 30 (5.2) |
|  | Dissatisfying | 29 (5.0) |
|  | Rather dissatisfying | 77 (13.3) |
|  | Rather satisfying | 72 (12.4) |
|  | Satisfying | 130 (22.3) |
|  | Very satisfying | 245 (42.1) |
| Physical health (LISAT3) | Very dissatisfying | 48 (8.3) |
|  | Dissatisfying | 68 (11.7) |
|  | Rather dissatisfying | 236 (40.5) |
|  | Rather satisfying | 83 (14.3) |
|  | Satisfying | 60 (10.3) |
|  | Very satisfying | 88 (15.1) |
| Psychological health (LISAT4) | Very dissatisfying | 30 (5.2) |
|  | Dissatisfying | 63 (10.9) |
|  | Rather dissatisfying | 107 (18.4) |
|  | Rather satisfying | 152 (26.1) |
|  | Satisfying | 149 (25.6) |
|  | Very satisfying | 82 (14.1) |
| Family life (LISAT5) | Very dissatisfying | 19 (3.3) |
|  | Dissatisfying | 55 (9.5) |
|  | Rather dissatisfying | 145 (24.9) |
|  | Rather satisfying | 89 (15.3) |
|  | Satisfying | 101 (17.4) |
|  | Very satisfying | 174 (29.9) |
| Vocational situation (LISAT6) | Very dissatisfying | 95 (16.3) |
|  | Dissatisfying | 108 (18.6) |
|  | Rather dissatisfying | 181 (31.1) |
|  | Rather satisfying | 33 (5.7) |
|  | Satisfying | 97 (16.7) |
|  | Very satisfying | 69 (11.9) |
| Leisure situation (LISAT7) | Very dissatisfying | 20 (3.5) |
|  | Dissatisfying | 41 (7.1) |
|  | Rather dissatisfying | 143 (24.6) |
|  | Rather satisfying | 145 (24.9) |
|  | Satisfying | 159 (27.3) |
|  | Very satisfying | 75 (12.9) |
| Sexual life (LISAT8) | Very dissatisfying | 21 (3.6) |
|  | Dissatisfying | 43 (7.4) |
|  | Rather dissatisfying | 129 (22.2) |
|  | Rather satisfying | 103 (17.7) |
|  | Satisfying | 180 (30.9) |
|  | Very satisfying | 107 (18.4) |
| Contacts with friends and acquaintances (LISAT9) | Very dissatisfying | 4 (0.7) |
|  | Dissatisfying | 7 (1.2) |
|  | Rather dissatisfying | 45 (7.8) |
|  | Rather satisfying | 106 (18.2) |
|  | Satisfying | 192 (33.0) |
|  | Very satisfying | 229 (39.3) |
| Partnership relation (LISAT10) | Very dissatisfying | 1 (0.2) |
|  | Dissatisfying | 30 (5.2) |
|  | Rather dissatisfying | 144 (24.7) |
|  | Rather satisfying | 123 (21.1) |
|  | Satisfying | 161 (27.7) |
|  | Very satisfying | 124 (21.3) |
| Life as a whole (LISAT11) | Very dissatisfying | 30 (5.2) |
|  | Dissatisfying | 85 (14.6) |
|  | Rather dissatisfying | 137 (23.5) |
|  | Rather satisfying | 84 (14.5) |
|  | Satisfying | 147 (25.3) |
|  | Very satisfying | 100 (17.2) |
| **Patient health questionnaire (PHQ)** | | |
| Little interest or pleasure in doing things (PHQ1) | Not at all | 514 (88.2) |
|  | Several days | 22 (3.8) |
|  | More than half the days | 33 (5.7) |
|  | Nearly every day | 14 (2.4) |
| Feeling down, depressed, or hopeless (PHQ2) | Not at all | 417 (71.6) |
|  | Several days | 65 (11.2) |
|  | More than half the days | 79 (13.6) |
|  | Nearly every day | 22 (3.8) |
| Trouble falling or staying asleep, or sleeping too much (PHQ3) | Not at all | 450 (77.2) |
|  | Several days | 69 (11.9) |
|  | More than half the days | 50 (8.6) |
|  | Nearly every day | 14 (2.4) |
| Feeling tired or having little energy (PHQ4) | Not at all | 493 (84.6) |
|  | Several days | 49 (8.4) |
|  | More than half the days | 32 (5.5) |
|  | Nearly every day | 9 (1.6) |
| Poor appetite or overeating (PHQ5) | Not at all | 525 (90.1) |
|  | Several days | 26 (4.5) |
|  | More than half the days | 21 (3.6) |
|  | Nearly every day | 11 (1.9) |
| Feeling bad about yourself—or that you are a failure or have let yourself or your family down (PHQ6) | Not at all | 534 (91.6) |
|  | Several days | 24 (4.2) |
|  | More than half the days | 18 (3.1) |
|  | Nearly every day | 7 (1.2) |
| Trouble concentrating on things, such as reading the newspaper or watching television (PHQ7) | Not at all | 512 (87.9) |
|  | Several days | 32 (5.5) |
|  | More than half the days | 34 (5.9) |
|  | Nearly every day | 5 (0.9) |
| Moving or speaking so slowly that other people could have noticed? Or the opposite (PHQ8) | Not at all | 524 (89.9) |
|  | Several days | 27 (4.7) |
|  | More than half the days | 19 (3.3) |
|  | Nearly every day | 13 (2.3) |
| Thoughts that you would be better off dead or of hurting yourself in some way (PHQ9) | Not at all | 547 (93.9) |
|  | Several days | 26 (4.5) |
|  | More than half the days | 7 (1.2) |
|  | Nearly every day | 3 (0.6) |
| **Diabetes symptoms severity (DSS)** | | |
| First symptom (DSS1) | No first symptom | 23 (4.0) |
|  | No problem | 244 (41.9) |
|  | Low | 240 (41.2) |
|  | Moderate | 63 (10.9) |
|  | High | 13 (2.3) |
| Second symptom (DSS2) | No second symptom | 97 (16.7) |
|  | No problem | 158 (27.1) |
|  | Low | 257 (44.1) |
|  | Moderate | 70 (12.1) |
|  | High | 1 (0.2) |
| Third symptom (DSS3) | No third symptom | 231 (39.7) |
|  | No problem | 97 (16.7) |
|  | Low | 194 (33.3) |
|  | Moderate | 56 (9.7) |
|  | High | 5 (0.9) |
| Fourth symptom (DSS4) | No fourth symptom | 379 (65.1) |
|  | No problem | 40 (6.9) |
|  | Low | 114 (19.6) |
|  | Moderate | 47 (8.1) |
|  | High | 3 (0.6) |

Note: µ=Mean.
